# Neuropsychiatric Symptoms in a p16-Positive Tongue Carcinoma Patient: An Unexpected Diagnosis

**DOI:** 10.7759/cureus.28256

**Published:** 2022-08-22

**Authors:** Mohamed Iliyas Sultan Abdul Kader, Lee Suk Sian, Abd Razak Ahmad, Nurulwafa Hussain, Arnil George Sirimanne, Mohd Razif Mohamad Yunus

**Affiliations:** 1 Department of Otorhinolaryngology - Head and Neck Surgery, Hospital Melaka, Melaka, MYS; 2 Department of Otorhinolaryngology - Head and Neck Surgery, Faculty of Medicine, Universiti Kebangsaan, Kuala Lumpur, MYS; 3 Department of Psychiatry, Hospital Melaka, Melaka, MYS; 4 Department of Oncology, National Cancer Institute, Wilayah Perseketuan Putrajaya, MYS; 5 Department of Otorhinolaryngology - Head and Neck Surgery, Universiti Kebangsaan Malaysia Medical Centre, Kuala Lumpur, MYS

**Keywords:** tongue carcinoma, neuropsychiatry, chemotherapy, acute mania, 5-fluorouracil

## Abstract

Differential diagnosis of neuropsychiatric symptoms in a patient with an underlying malignancy is exhaustive. 5-fluorouracil (5-FU) is one of the most widely used chemotherapy agents and it is often used as the first-line regimen in head and neck malignancies. We present a case of an elderly female with an underlying locally advanced p16-positive squamous cell carcinoma of the tongue who presented with manic symptoms for one week after cycle 2 of chemotherapy. Multidisciplinary management by otorhinolaryngologists with psychiatrist and oncologist led to the cessation of 5-FU, administration of antipsychotics, and replacement with a different chemotherapy agent, leading to complete resolution of manic symptoms. Possible mechanisms of the 5-FU-induced manic episode with its treatment are discussed in this report.

## Introduction

5-fluorouracil (5-FU) is an anti-metabolite that is commonly used for breast, colorectal, head and neck, pancreas, and stomach cancers. 5-FU exerts its anti-neoplastic effects by inhibiting the synthesis of DNA and RNA by inhibiting thymidylate synthase [1]. The most common side effects include gastrointestinal symptoms (nausea, vomiting, mucositis, diarrhoea, and anorexia), effects of myelosuppression (neutropenia, thrombocytopenia), alopecia, dermatological effects, skin discoloration, and metallic taste. Rare but severe toxicity, including cardiotoxicity and neurotoxicity, has also been reported [2]. 

When present, the commonly reported neurotoxicity of 5-FU includes upper motor neuron signs, cerebellar ataxia, somnolence, and signs of organic brain syndrome [3]. Neuropsychiatric symptoms of mania are rarely associated with 5-FU usage.

We present a rare case of 5-FU induced bipolar disorder, manic episode in a lady with no prior history of psychiatric illness. The patient was on intravenous 5-FU and cisplatin for her locally advanced tongue cancer (p16 positive) and developed acute manic symptoms following the second cycle of chemotherapy. Diagnosis and management in literature are discussed.

This article was previously presented as a meeting abstract at the 13th Malaysian International ORL-HNS (Otolaryngology-Head and Neck Surgery) Congress in conjunction with the 7th Conference of the Asian Society of Head and Neck Oncology (ASHNOKL 2021) on June 1, 2021.

## Case presentation

A 61-year-old lady was diagnosed with locally advanced tongue cancer cT4N1M0 of moderately differentiated squamous with p16 positivity after a year of progressive enlarging tongue mass and odynophagia. Apart from diabetes on metformin and insulin, the patient did not have any other comorbid conditions. The patient does not consume alcohol. She had no previous history of psychiatric illness and had no family history of psychiatric conditions. Chemotherapy of intravenous 5-fluorouracil 750 mg/m2 and cisplatin 75 mg/m2, 3 weekly, were commenced with premedication IV dexamethasone 8 mg. The first two cycles of chemotherapy were uneventful and the patient responded well to chemotherapy clinically with significant tumour bulk reduction and regained normal oral feeding. A week after the second cycle, the patient rapidly developed an elevated mood, insomnia, mood-congruent delusion of being possessed by a deity who cured her disease, visual hallucination of seeing the deity, and being aggressive towards family members. On examination, the patient appeared to be energetic, elevated mood, and increased speech with tangentiality. She was orientated to time, place, and person, had good eye contact, but had disorganized and pressured speech. Neurological and cardiovascular examinations were unremarkable.

**Figure 1:**
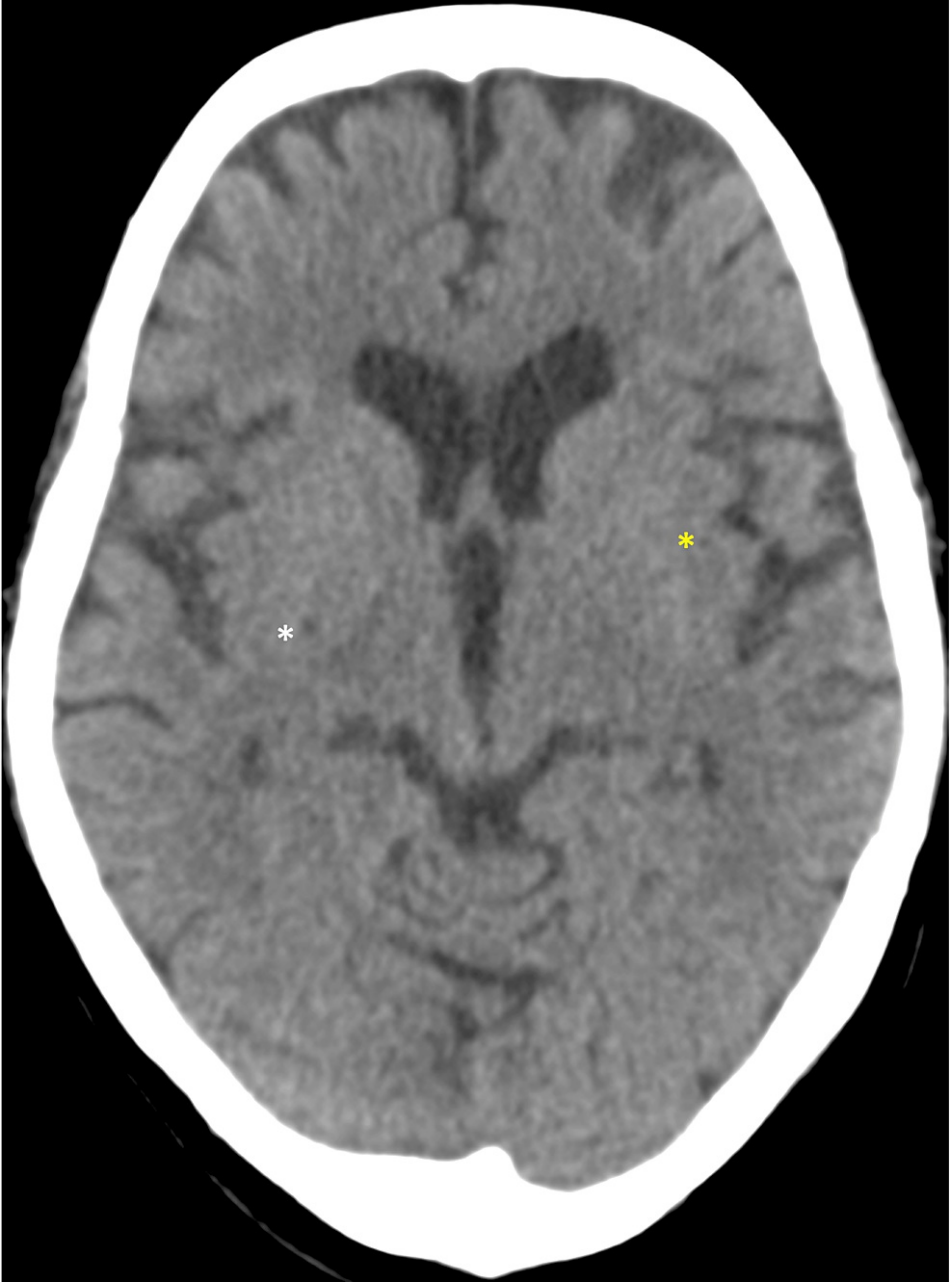
Contrast-enhanced computed tomography (CECT) of brain White asterisk (*) shows old lacunar infarcts in the right lentiform nucleus; yellow * shows old infarcts in the left external capsules.

Electrocardiogram and urine toxicology were normal. Glucometer showed an elevated blood sugar level of 22 mmol/L, which was attributable to her missed diabetic medications. Blood ammonia was slightly elevated at 53 μmol/L (normal 1-47 μmol/L). Blood gases, serum ketone, serum osmolarity, thyroid function test, liver function test, HIV, hepatitis B, C, syphilis, urea and electrolytes, and urine drug toxicology were otherwise normal. Contrast-enhanced computed tomography (CECT) brain (Figure 1) showed old lacunar infarcts in the right lentiform nucleus and left external capsules in the background of a generalized atrophic cerebrum. There was no focal enhancing brain parenchyma lesion, no abnormal leptomeningeal enhancement, no cerebral edema and there was normal grey-white matter differentiation. A diagnosis of 5-fluorouracil-induced bipolar disorder, manic episode (ICD-10 F19.94) was made. 

In the ward, she was started on oral haloperidol 5 mg at night and oral diazepam 10 mg at night. The patient was hostile, disturbing other patients, argumentative, and not cooperative, due to which she had to be temporarily restrained. Her agitated behaviour was stabilized with a parenteral tranquilizer of intravenous diazepam 5 mg and intramuscular haloperidol 5 mg, and then a 12-hourly dosing of oral haloperidol 5 mg and oral diazepam 10 mg. The patient subsequently progressed well, with resolved symptoms of mania. She was able to wean off haloperidol and diazepam on day 3 of admission and upon follow-up a week later, her mental state examination remained normal with only increased speech; no other symptoms were present. In one month, the patient was completely normal with no permanent sequelae noted. The patient was restarted with IV gemcitabine 1000 mg/m2 and cisplatin 35 mg/m2 for two cycles, followed by concurrent radiotherapy with cisplatin 30 mg/m2. The patient planned for a total of 35 fractions, however, she only completed 28 fractions and refused further radiotherapy.

## Discussion

Diagnosis of 5-FU-related bipolar disorder, manic episode is one of exclusion. The possible differential diagnoses for our patient at presentation of psychiatric symptoms include brain metastasis secondary to tongue malignancy, electrolyte abnormalities, diabetic ketoacidosis, infectious, substance abuse, functional psychotic illness, and adverse effect by concomitant medications (cisplatin and dexamethasone). Detailed history and exhaustive investigations were negative for all the other possible diagnoses. Lumbar puncture was not performed as there were no symptoms of meningitis (absent fever and neck stiffness, Kernig & Brudzinski sign negative). Steroids are known to cause mood disorders; their effect is dose-dependent but the likelihood in our patient was extremely unlikely as our patient only received two doses of IV dexamethasone 8 mg prior to chemotherapy [4]. The rapid and complete disappearance of manic symptoms was observed with the discontinuation of 5-FU, and the administration of anti-psychotic medication further supported our diagnosis of 5-FU-induced bipolar disorder manic episode. Imaging modalities often reveal leukoencephalopathy in patients receiving 5-FU with neurotoxicity [5]. However, in our patient CECT brain did not reveal leukoencephalopathy.

Acute psychosis was previously reported in a patient with colon carcinoma receiving 5-FU, oxaliplatin, and bevacizumab [6]. Another patient with breast carcinoma also developed a manic episode when she was receiving 5-FU, epirubicin, and cyclophosphamide [7]. The pathophysiology of 5-FU-induced manic episodes is not fully understood. However, several mechanisms have been postulated. Neurotoxicity can be caused by ammonia, the end product of 5-FU, and fluoroacetate, a by-product of 5-FU. The increase in ammonia inhibits adenosine triphosphate (ATP)-producing Kreb's cycle, which affects the urea cycle [8]. Our patient had slightly elevated ammonia levels. The second proposed theory is the adverse effect of 5-FU, which decreases the thiamine pyrophosphate (TPP), an active form of vitamin B1. This theory is due to the fact that 5-FU-induced neurotoxic effects are similar to Wernicke-Korsakoff syndrome, in which patients develop ataxia, nystagmus, mental confusion, and cognitive changes [9].

The majority of the administered 5-FU is catabolized by an enzyme called dihydropyrimidine dehydrogenase (DPD), which is distributed in the liver, gastrointestinal mucosa, and peripheral lymphocytes [10]. Incidence of DPD deficiency estimated to be 0.1% to 2.7% in cancer patients [11]. Therefore, if a patient with DPD deficiency was treated with fluorouracil, the patient can develop serious toxicity. Cordier et al reported two patients who developed neurotoxicity after their first course of 5-FU. They retrospectively found that these patients were suffering from profound DPD deficiency [12]. Several authors recommend screening for DPD deficiency before 5-FU treatment, however, it is not practical to obtain levels of DPD for all patients because of its low incidence [13]. Furthermore, the enzyme assay is complicated and not readily available [14]. We did not test for DPD deficiency in our patient. As even if it is present, discontinuation of 5-FU with conservative treatment will result in improvement of symptoms.

## Conclusions

The otorhinolaryngology team should be aware of 5-FU-induced neuropsychiatric symptoms despite their being a rare occurrence. For this patient, a prompt multidisciplinary approach, including cessation of 5-FU usage, co-management with psychiatric colleagues by administering antipsychotics, and an alternative chemotherapy agent to treat the primary malignancy by consulting with the oncology team led to the reversal of symptoms.
